# Impact of Empagliflozin on mRNA Levels of Cardiotrophin-1 and GDF-15 in Patients with Type 2 Diabetes and ST-Segment Elevation Myocardial Infarction Undergoing PCI: A Double-Blind Pilot Trial

**DOI:** 10.34172/jcvtr.33675

**Published:** 2026-06-30

**Authors:** Reza Rastgoo, Mahnaz Yousefi Ramandi, Fereshteh Ebrahimi, Reza Madadi, Hamid Khederlou, Ali Ramazani

**Affiliations:** ^1^Department Pharmaceutical Biotechnology, School of Pharmacy, Zanjan University of Medical Sciences, Zanjan, Iran; ^2^Department of Cardiology, Zanjan University of Medical Sciences, Zanjan, Iran

**Keywords:** Empagliflozin, Cardiotrophin-1, Growth differentiation factor-15, Diabetes mellitus, Blood sugar, Myocardial infarction

## Abstract

**Introduction:**

Given the proven cardiovascular benefits of Empagliflozin (EM), we aimed to investigate the effect of Empagliflozin on Cardiotrophin-1 (CTF-1) and growth differentiation factor-15 (GDF-15) expressions in circulating monocytes in type 2 diabetic patients after MI.

**Methods:**

This randomized, double-blind, placebo-controlled study included 33 patients referred to Ayatollah Mousavi Hospital in Zanjan between 5 of September to 25 of December, 2024. Eligible patients with type 2 diabetes and MI who underwent primary percutaneous coronary intervention (PCI) were randomly assigned (17:16) to receive either 10 mg of EMPA daily or placebo for 12 weeks. Target genes expression levels were quantified by real time PCR, and data were analyzed by SPSS version 26.

**Results:**

Thirty-three patients were included in this study (mean age: 61.27±9.07, 66% male), mean diabetes duration was longer in intervention group (7.82±5.13) compared to placebo group (3.75±2.74), hypertension was the most common comorbidity (57%). CTF-1 expression in the intervention group was 0.3±0.28 and in placebo group was 5.66±6.52. GDF-15 expression in the intervention group was 0.198±0.22 and in placebo group was 1.66±1.04. EMPA significantly reduced expression of CTF-1 (13-fold) (*P*=0.003) and GDF-15 (7-fold) (*P* ˂ 0.0001). The CTF-1 and GDF-15 expression reduction were not associated with any other clinical variables. Significant reduction observed in LDL (*P*=0.045) and triglycerides (*P*=0.013) after intervention.

**Conclusion:**

EMPA significantly reduced the expression of CTF-1 and GDF-15 in type 2 diabetic, post MI patients, which are associated with atherosclerosis, and suggest that potential anti-atherosclerotic effect of EMPA mediated through modulation of GDF-15 and CTF-1 gene expression.

## Introduction

 Myocardial infarction (MI) is one of the most severe acute kind of cardiovascular disease.^1^ MI most commonly results from the rupture of vulnerable atherosclerotic plaques and subsequent coronary artery thrombosis.^2,3^ Subsequent to a myocardial infarction (MI), atherosclerotic plaques may indeed experience accelerated growth and increased complexity, thereby heightening the risk of another MI.^4^ Moreover, MI is influenced by both traditional and novel risk. Traditional risk factors include type 2 diabetes mellitus (T_2_DM), obesity, dyslipidemia, family history of heart disease and novel risk factors such as chronic inflammation, autoimmune diseases,^5^ thrombopoietin^6^ and cardiac biomarkers like cardiotrophin-1 (CTF-1).^7^

 T_2_DM is a chronic metabolic disorder^8^ that accounts for about 90% of diabetic population in world.^9^ Macrovascular complications of T_2_DM include coronary arteries, peripheral arteries and cerebrovasculature, with an annual incidence of 1.4 - 4.7% of patients.^10,11^ There are different oral medicines for T_2_DM treatment management such as metformin, sulfonylureas, sodium-glucose co-transporter 2 inhibitors (SGLT2Is), dipeptidyl peptidase 4 inhibitors, and the choice of treatment depends on patient’s clinical conditions. Empagliflozin (EMPA) is a member of SGLT2I that used in T_2_DM treatment. EMPA increases urinary glucose excretion, causes glucose reduction, lowers body weight and blood pressure.^12^ EMPA is utilized in the management of T2DM, as well as for individuals who have concurrent cardiovascular conditions, particularly heart failure.^12-16^ Furthermore, through an unidentified process, EMPA may diminish oxidative stress and the generation of free radicals. This indicates that the protective benefits of EMPA could go beyond its established mechanisms, such as regulating blood sugar, and may engage in a more essential interaction with the body’s antioxidant defenses.^14^

 Biomarkers in body are used for diagnosis, prognosis, prediction and monitoring therapeutic responses.^17^ Among cardiovascular and diabetic biomarkers, Galectin-3, cardiotrophin-1 (CTF-1) and growth differentiation factor-15 (GDF-15) are of particular interest.^18^ Evidence indicates that EMPA can modulates CTF-1 and GDF-15 expression levels.^19-21^ CTF-1 is one of cardiovascular and diabetic biomarkers, a member of interlukin-6 cytokine family^22^ that can exert its effects by binding to gp130 and Leukemia Inhibitory Factor Receptor (LIFR).^23^ CTF-1 is produced by myocardial cells of the heart^22^, vascular endothelial cells, macrophages, and adipose tissue.^24,25^ Its expression varies among these tissues; instead, it demonstrates functions that are specific to each tissue. In the heart, increased CTF-1 expression is associated with cardiac hypertrophy,^23,26^ in adipose tissue, it promotes lipolysis and reduce insulin resistance ^27^ and in vascular endothelial cells, atherosclerotic plaques, and macrophages it initiates inflammatory processes. CTF-1 stimulate expression of interleukin-6, interleukin-1β and *TNF–a* in monocytes,^26-30^ enhanced monocytes adhesion and migration into atherosclerotic plaques^30^ and has been shown in ApoE^–/–^ mice to be highly expressed in macrophages, that accelerates plaques progression.^24^ GDF-15 biomarker also known as macrophage inhibitory cytokine-1 (MIC-1), belongs to the transforming growth factor beta (TGF-β) superfamily.^31^ GDF-15 is generally found at low concentrations in the majority of tissues under standard physiological circumstances. ^21^ Nevertheless, its expression is markedly increased in response to stress and tissue injury. Although the placenta serves as the main source of elevated GDF-15 levels during pregnancy, it is also produced in higher amounts in several pathological states, particularly those involving endothelial cells, vascular smooth muscle cells, and adipose tissue.^32^ Higher concentration of GDF-15 associated with arterial fibrillation risk^33^ and mortality in chronic heart failure patients.^31^ GDF-15 has been shown to enlarge atherosclerotic plaques and increase plaque vulnerability.^34^

 In this study we aimed to investigate potential anti-atherosclerosis effect of Empagliflozin by evaluating its impact on CTF-1 and GDF-15 expression in peripheral blood in patients with T_2_DM after MI undergoing primary PCI.

## Material and Methods

###  Study design

 The trial was a randomized, double-blind, placebo-controlled trial, in which T_2_DM patients post-ST-segment elevation MI (STEMI) underwent primary percutaneous coronary intervention (PCI) were randomly assigned to EMPA 10 mg daily or matching placebo (1:1) for 12 weeks between September 5 to December 25, 2024. Our study was approved by the Ethic Committee of Zanjan University of Medical Sciences (Ethical code: IR.ZUMS.REC.1403.154), and the protocol was prospectively registered on the Iranian Registry of Clinical Trials (www.IRCT.ir, IRCT code: IRCT20241106063619N1).

 The inclusion criteria of patients were T_2_DM patients with HbA1c between 6.5-8% treated with metformin, sulfonylurea, and other anti-diabetic agents or combination of all of these medicines, and patients with typical chest pain during the 12 hours prior to assessment that lasting longer than 30 minutes, along with an ST-segment elevation above 0.1 mV in 2 anatomically contiguous leads or ≥ 2 mm in leads V2 and V3 or new left bundle branch block in ECG. The exclusion criteria was cardiogenic shock, hypoglycemia, diabetic ketoacidosis, History of Coronary Artery Bypass Surgery (CABG), type 1 diabetes mellitus, sever liver failure, history of any cancer and chemotherapy, history of severe allergic reaction to Empagliflozin or its formulation components, Severe renal failure (eGFR < 30 mL/minute/1.73 m2), end-stage renal disease (ESRD), or dialysis, hypovolemia, any inflammatory under treatment disease, history heart failure (HF).^35,36^ After screening medical records 55 patients met inclusion criteria. Among these, 45 patients were randomized (1:1) to receive either Empagliflozin 10 mg or matching placebo; during treatment period, 10 patients (five from each group) were excluded during follow-up (Figure 1).

**Figure 1:**
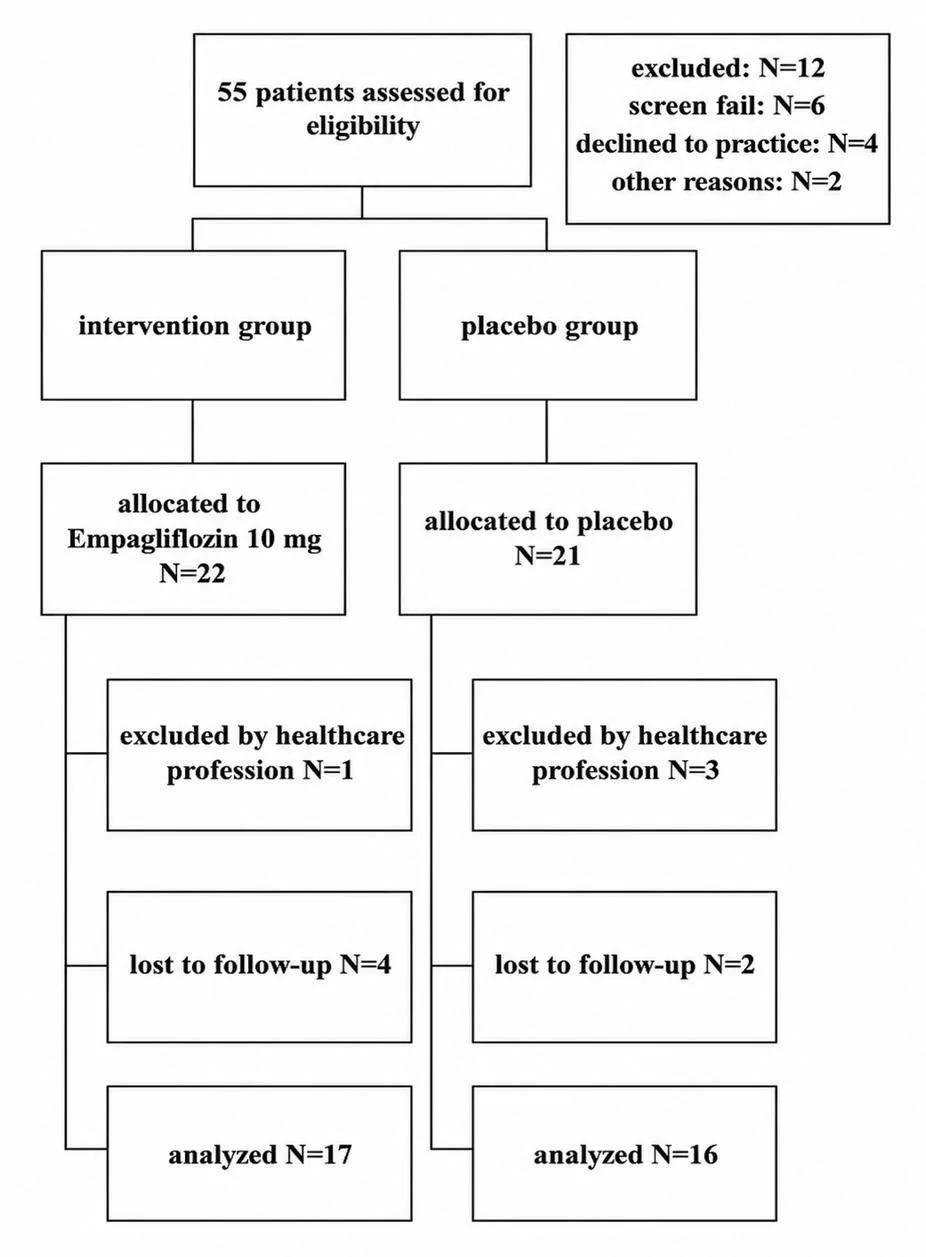
Flowchart of project study

###  Intervention, randomization and follow-up

 Insulin therapy was started for all patients to manage blood glucose, and metformin was added if appropriate. To maintain optimal blood sugar level, patients’ dietary recommendation during patient’s nutritional counseling, the patient’s blood sugar medicines are adjusted, and if necessary, insulin added to treatment, and patient’s fasting blood sugar maintained at 80 – 130 mg/dL and 2 hours after meal sugar maintained at 140 – 180 mg/dL. Patients received metformin, sulfonylurea, and other anti-diabetic agents or combination of all of these were included. Those treated with older-generation of sulfonylurea, they were transitioned to newer-generation of sulfonylurea for better glycemic control. Patients were randomly assigned to receive either EMPA (10 mg tablets from Dr. Abidi Pharmaceuticals, Tehran, Iran) daily for 12 weeks, or matching placebo. EMPA was started after 24 hours of hospitalization and reperfusion, if hemodynamics were stable and the exclusion criteria were ruled out. Randomization was conducted by a third-party collaborator using coded assignment. A set of odd and even codes was drawn without replacement, ensuring unbiased assignment of drug and placebo. Peripheral blood sample was collected before of initiating of the EMPA, and again at the end of 12-week intervention. Patients returned one month and then 3 months after PPCI to assess compliance with medication use, review drug side effects, and also control cardiovascular risk factors and EF assessment.

###  Gene expression analyzes

 Five ml of patients’ blood sample was centrifuged and buffy coat was separated and stored in -80 °C until use. Total RNA was extracted by Sambio^TM^ RNA extraction kit (Sambio^TM^, China). The quantity of extracted RNA was analyzed by Eppendorf BioPhotometer Plus (Eppendorph, Germany). Based on concentration, RNA was adjusted for 1 µg and cDNA was synthesized by Samzol kit (sam020c, China). Gradient PCR was used to optimize annealing temperatures of primers for target and housekeeping (GAPDH) genes (60 °C for CTF-1 and 59 °C for both GDF-15 and GAPDH). Expression levels of target and housekeeping gene were quantified by Real-time PCR by ABI 7300 Real-Time PCR System (Applied Biosystems) with specific primers (Table 1), and analyzed by using 2^-∆∆CT^ method. The qRT-PCR reactions were performed using Sambio^TM^ SYBR qPCR Master Mix (Sambio^TM^, China) according to the manufacturer’s protocol. The reaction mixture consisted of 500 ng of cDNA template, 10 μL of 2 × Sambio^TM^ SYBR qPCR Master Mix, 0.4 μL of forward primer (10 μM), and 0.4 μL of reverse primer (10 μM) in a final reaction volume of 20 μL. A non-template negative control were included for each primer set to confirm the absence of genomic DNA and contamination in the reactions. The qRT-PCR protocol included an initial step of 95 °C for 4 min, followed by 40 cycles of 95 °C for 20 s and then annealed at 59 °C (GDF-15, GAPDH) and 60 °C for CTF-1) for 20 s, extension at 72 °C for 40 s.

**Table 1:** Primer sequence (5′ to 3′) used for target amplification

**Gene name**	**Forward primer**	**Reverse primer**	**PCR product length (bp)**
CTF-1	CACTTGGAGGCCAAGATCC	TCTCCCTGGAGCTGCACAT	70
GDF-15	GTTAGCCAAAGACTGCCACTG	CCTTGAGCCCATTCCACA	106
GAPDH	CTGCACCACCAACTGCTTAG	GTCTTCTGGGTGGCAGTGAT	108

###  Statistical analyses

 All Data are expressed as mean values, and statistical analyses were performed by SPSS version 26 (IBM, USA). Normality was assessed by the Shapiro-Wilks test. For normally distributed data, independent *t *test (between groups) and paired *t* test (within group) were used. For non-normal distributions, Mann-Whiteny U test (between groups) and Wilcoxon signed-rank test (within groups) were used. Differences were considered statistically significant at *P*-value less than 0.05.

## Results

 The mean age of participants was 61.27 ± 9.07, and 22 (66%) of patients were male. Comparing an intervention group to a placebo group, mean duration of diabetes was longer in intervention group (7.82 ± 5.13) than in the placebo group (3.75 ± 2.74). Hypertension was present in 19 (57%) patients, ischemic heart disease present in 7 (21%), and dyslipidemia in 17 (51%) patients. The mean ejection fraction was 37.88 ± 8.104. PCI was performed in 6 (18%), and 15 (45%) were smokers. The most common culprit vessels were the right coronary artery (RCA) in 15 (45%) patients, and left anterior descending artery (LAD) in 14 (42%) patients. RCA occlusion typically results in an inferior MI, while LAD occlusion typically led to extensive anterior infarction with greater prognostic impact (Table 2).

**Table 2:** Baseline characteristics, laboratory, echocardiographic, and angiographic data

**Variable**	**Placebo group** **N (%), mean (std)**	**Intervention group** **N (%), mean (std)**	***P*** ** value**
Gender (male)	13 (81.3%)	9 (52.9%)	0.141
Diabetes duration	3.75 (2.74)	7.82 (5.13)	0.022*
History of hypertension	8 (50%)	11 (64.7%)	0.407
History of ischemic heart disease	2 (12.5%)	5 (29.4%)	0.398
History of hyperlipidemia	5 (31.3%)	12 (70.6%)	0.038*
Smoking	10 (62.5%)	5 (29.4%)	0.084
History of PCI	2 (12.5%)	4 (23.5%)	0.656
LDL (mg/dL)	98.06 (± 26.144)	91.76 (± 20.398)	0.612
HDL (mg/dL)	39.63 (± 6.5)	37.35 (± 7.407)	0.187
FBS (mg/dL)	174.94 (± 61.518)	218.76 (± 85.896)	0.144
Triglyceride (mg/dL)	147.5 (± 52.004)	133.24 (± 48.763)	0.551
Cholesterol (mg/dL)	170.81 (± 52.439)	155.94 (± 31.558)	0.46
BM (kg/m^2^)	27.03 (± 2.40)	26.776 (± 2.3104)	0.979
Systolic blood pressure (mmHg)	128.44 (± 26.377)	122.35 (± 18.844)	0.596
Diastolic blood pressure (mmHg)	79.69 (± 14.659)	77.65 (± 11.608)	0.793
GFR (ml/min/1.73m^2^)	73.63 (± 10.106)	65.29 (± 23.9.19)	0.256
Heart failure severity (Based on EF)			
Mild	6 (37.5%)	5 (29.4%)	0.656
Moderate	7 (43.8%)	8 (47.7%)	
Severe	3 (18.8%)	4 (23.5%)	
TIMI-before-PCI			
0	9 (56.3%)	10 (58.8%)	1
1	7 (43.8%)	7 (41.2%)	
2	0 (0%)	0 (0%)	
3	0 (0%)	0 (0%)	
TIMI-after-PCI			
0	0 (0%)	0 (0%)	0.688
1	0 (0%)	0 (0%)	
2	4 (25%)	3 (17.6%)	
3	12 (75%)	14 (82.4%)	
Culprit vessel			
LAD	6 (37.5%)	8 (47.1%)	0.382
LCX	1 (6.3%)	3 (17.7%)	
RCA	9 (56.3%)	6 (35.3%)	

 There were no significant differences in clinical and laboratory characteristics between two groups. Both groups showed reduction in body mass index (BMI), fasting blood glucose (FBS), low density lipoprotein (LDL), cholesterol, triglyceride and high-density lipoprotein (HDL) levels, but LDL (*P* = 0.045) and triglyceride (*P* = 0.006) decreased more in the intervention group (Table 3 and 4).

**Table 3:** Change in clinical variables and laboratory measurements after intervention between placebo and intervention group

**Variable**	**Before intervention**		***P*** **-value**	**After intervention**		***P*** **-value**
	**placebo**	**intervention**		**placebo**	**intervention**	
LDL (mg/dL)	98.06 (± 26.144)	91.76 (± 20.398)	0.612	66.13 (± 10.601)	58.88 (± 9.347)	0.045*
HDL (mg/dL	39.63 (± 6.5)	37.35 (± 7.407)	0.187	50.44 (± 10.347)	56.53 (± 11.375)	0.138
FBS (mg/dL	174.94 (± 61.518)	218.76 (± 85.896)	0.144	142.56 (± 36.425)	118.29 (± 22.588)	0.059
Triglyceride (mg/dL	147.5 (± 52.004)	133.24 (± 48.763)	0.551	100.38 (± 23.174)	74.29 (± 26.224)	0.006**
Cholesterol (mg/dL	170.81 (± 52.439)	155.94 (± 31.558)	0.46	125.69 (± 21.291)	130.41 (± 22.344)	0.539
BMI kg/m^2^	27.03 (± 2.4025)	26.776 (± 2.3104)	0.979	26.681 (± 2.3022)	26.335 (± 2.3208)	0.908
Systolic blood pressure (mmHg)	128.44 (± 26.377)	122.35 (± 18.844)	0.596	121.88 (± 8.539)	122.06 (± 8.849)	0.923
Diastolic blood pressure (mmHg)	79.69 (± 14.659)	77.65 (± 11.608)	0.793	77.19 (± 7.739)	74.41 (± 8.269)	0.375
GFR (ml/min/1.73m^2^)	73.63 (± 10.106)	65.29 (± 23.919)	0.256	75.56 (± 20.043	66.18 (± 18.375)	0.159

**Table 4:** Change in clinical variables and laboratory measurements after intervention in each group before and after intervention

**Variable**	**Before intervention**		***P*** **-value**	**After intervention**		***P*** **-value**
	**placebo**	**intervention**		**placebo**	**intervention**	
LDL (mg/dL)	98.06 (± 23.144)	66.13 ((± 10.601)	< 0.001***	91.76 ((± 20.398)	58.88 ((± 9.347)	< 0.001***
HDL (mg/dL)	39.63 (± 6.5)	50.44 ((± 10.347)	< 0.001***	37.35 ((± 7.407)	56.53 ((± 11.357)	< 0.001***
FBS (mg/dL)	174.94 (± 61.518)	142.56 ((± 36.245)	< 0.001***	218.76 ((± 85.896)	118.29 ((± 22.588)	< 0.001***
Triglyceride (mg/dL)	147.5 (± 52.004)	100.38 ((± 23.174)	< 0.001***	133.24 ((± 48.763)	74.29 ((± 26.224)	< 0.001***
Cholesterol (mg/dL)	170.81 ((± 52.439)	125.69 ((± 21.291)	< 0.001***	155.94 ((± 31.558)	130.41 ((± 22.344)	< 0.001***
BMI kg/m^2^	27.03 (± 2.40)	26.681 ((± 2.30)	0.002**	26.776 ((± 2.31)	26.335 ((± 2.32)	0.003**
Systolic blood pressure (mmHg)	128.44 (± 26.37)	121.88 ((± 8.53)	0.422	122.35 ((± 18.84)	122.06 ((± 8.84)	0.886
Diastolic blood pressure (mmHg)	79.69 (± 14.65)	77.19 ((± 7.73)	0.371	77.65 ((± 11.60)	74.41 (± 8.26)	0.25
GFR (ml/min/1.73m^2^)	73.63 (± 10.109)	75.56 ((± 20.043)	0.096	65.29 ((± 23.919)	66.18 ((± 18.375)	0.969

 CTF-1 expression level was significantly lower (1-fold) in the intervention group 0.3 ± 0.28 compared to placebo group 5.66 ± 6.52 (*P* = 0.003). GDF-15 expression was also reduced (7-fold) in the intervention group 0.198 ± 0.22 compared to placebo group 1.66 ± 1.04 (*P* ˂ 0.0001). This reduction was not associated with other clinical variables (Figure 2).

**Figure 2:**
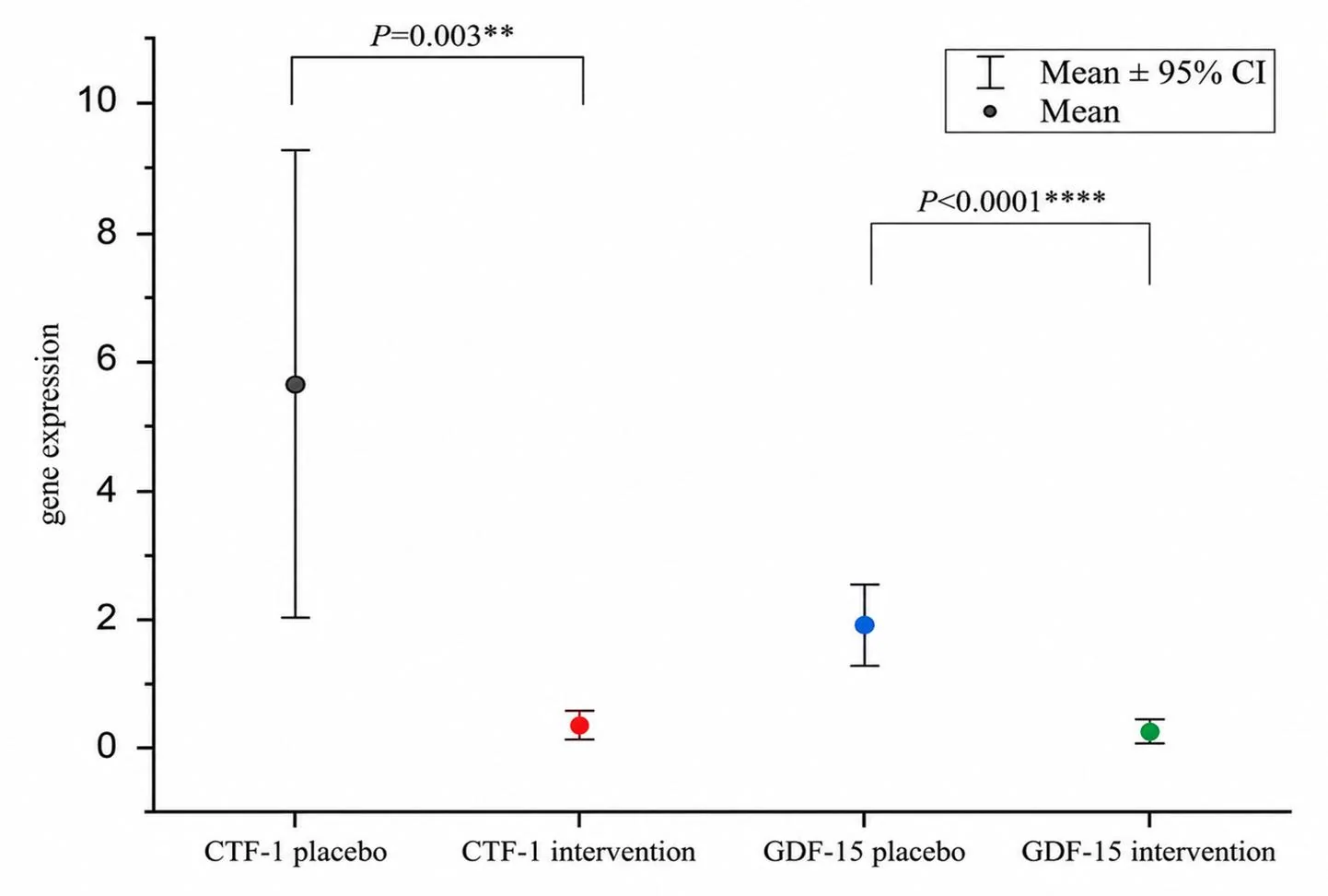
CTF-1 and GDF-15 gene expression, control level considered 1

## Discussion

 EMPA is a drug from the SGLT2 inhibitor class of drugs that is recognized as a novel drug for the treatment of T2DM. EMPA has been shown to significantly improve patients’ BMI, microalbuminuria, BUN, FBS, 2-hour postprandial blood glucose, HbA1c, triglycerides, cholesterol, and LDL (*P* < 0.05), while HDL levels improved without reaching statistical significance.^1^ In our study, LDL and triglyceride levels improved significantly (*P* < 0.05), while FBS levels improved, which was not statistically significant (*P* = 0.059). These findings confirm the potential clinical benefits of EMPA.

 EMPA is also known to have protective effects on CVD, including reducing major adverse cardiovascular events (MACE) in ischemic heart disease (IHD) patients, reducing mortality and hospitalization in heart failure (HF) patients, etc. These effects include decreased blood glucose levels, decreased oxidative stress, and positive effects on cytokines and chemokines.^2^ It also reduced the secretion of CTF-1 and GDF-15 proteins in human smooth muscle cells. CTF-1 and GDF-15 proteins are considered inflammatory factors.^3,4^ CTF-1 is highly expressed in cardiac muscle cells, cardiovascular cells, vascular epithelial cells, vascular smooth muscle cells, and macrophages.^5-7^ Chronic overexpression of this protein increases fatty acid oxidation, fibroblast growth, foam cell formation in the vascular intima, and atherosclerosis, while also increasing oxidative stress factors, cytokines, and chemokines (8). In the APOE^(-/-)^ mice model, it has been shown that CTF-1 protein affects the formation of atherosclerotic plaques by increasing macrophage migration, increasing expression of inflammatory factors, and increasing foam cell formation in the vascular intima. ^9,10^ CTF-1 is normally expressed at low levels in monocytes, and this biomarker is highly upregulated in macrophages of atherosclerotic plaques. Studies in APOE^(-/-)^ mice have shown a strong correlation between CTF-1 expression and the severity of atherosclerosis.^10^ GDF-15 is a stress-responsive biomarker that has been associated with various cardiovascular diseases, including atherosclerotic plaque formation, IHD, heart failure, various arrhythmias, hypertension, etc. ^11^ GDF-15 is expressed at high levels in myocardial cells under inflammatory conditions, and plasma levels of GDF-15 are increased accordingly. In patients with HF with reduced ejection fraction (HFrEF), GDF-15 is increased, which is accompanied by increases in high-sensitivity C-reactive protein and high-sensitivity troponin T.^19^

 Our study is the first to evaluate the effect of EMPA on peripheral blood CTF-1 and GDF-15 expression. In this study, we studied 33 type 2 DM patients with MI who underwent primary PCI. We found that treatment with EMPA 10 mg daily for 12 weeks resulted in a decrease in CTF-1 and GDF-15 expression in peripheral blood and peripheral circulating monocytes, suggesting a potential anti-atherosclerotic mechanism. Our data support that EMPA exerts its anti-atherosclerotic effects in part by reducing monocyte CTF-1 expression and potentially through mechanisms involving vascular smooth muscle cells. Interestingly, our data show that EMPA reduces the expression of GDF-15 in peripheral circulating monocytes, which is associated with the formation of atherosclerotic plaques. Consequently, EMPA may reduce the risk of cardiovascular disease by reducing the expression of GDF-15, which is associated with various types of cardiovascular disease.

## Conclusion

 In this study, we showed that EMPA reduced CTF-1 and GDF-15 expression in type 2 diabetic patients with MI who underwent primary PCI. Given the established roles of CTF-1 and GDF-15 as cardiovascular biomarker, their decreased expression suggests potential cardio-protective benefits of EMPA. Further large-scale studies are recommended to confirm the potential benefits of EMPA in managing atherosclerosis in patients after MI.

## Competing Interests

 There is no conflict of interest.

## Ethical Approval

 This study was approved by the Ethics Committee of Zanjan University of Medical Sciences (approval code. Ethical code: IR.ZUMS.REC.1403.154) and the protocol was prospectively registered on the Iranian Registry of Clinical Trials (www.IRCT.ir, IRCT code: IRCT20241106063619N1). Furthermore, all participants received and signed written informed consent.
